# Longitudinal analysis of body compositions following Roux-en-Y gastric bypass

**DOI:** 10.1007/s00423-026-03994-8

**Published:** 2026-02-21

**Authors:** Zubaidah Nor Hanipah, Gabriela de O. Lemos, Sophia Ramirez, Venkata Satya Naga Arun Kousik Dhulipala, Karteek Popuri, Mirza Faisal Beg, Shengping Yang, Philip R. Schauer, Vance L. Albaugh, Steven B. Heymsfield

**Affiliations:** 1 Metamor Institute, Pennington Biomedical Research Center, Louisiana State University, 6400 Perkins Rd., Baton Rouge, LA 70808 USA; 2 Department of Surgery, Faculty of Medicine and Health Science, University Putra Malaysia, Serdang, Selangor Malaysia; 3 Metabolism-Body Composition Laboratory, Pennington Biomedical Research Center, Louisiana State University, Baton Rouge, LA USA; 4 Laboratory of Nutrition and Metabolic Surgery, Department of Gastroenterology, University of São Paulo School of Medicine-FMUSP, São Paulo, SP Brazil; 5 Department of Computer Science, Memorial University of Newfoundland, St. John’s, NL Canada; 6 School of Engineering Science, Simon Fraser University, Vancouver, BC Canada; 7 Biostatistics Department, Pennington Biomedical Research Center, Louisiana State University, Baton Rouge, LA USA; 8 Department of Surgery, Louisiana Health Sciences Center, New Orleans, LA USA; 9 Our Lady of the Lake Regional Medical Center, Baton Rouge, LA USA

**Keywords:** Roux-en-Y gastric bypass, Skeletal muscle, Subcutaneous adipose tissue, Visceral adipose tissue, Computed tomography

## Abstract

**Background:**

Roux-en-Y gastric bypass (RYGB) is associated with substantial weight loss and improved obesity-related comorbidities. However, outcomes on body composition, particularly skeletal muscle (SM), visceral adipose tissue (VAT), and subcutaneous adipose tissue (SAT) remain inconsistent due to limitations in measurement techniques.

**Objective:**

Evaluate longitudinal changes in SM, VAT, and SAT volumes (cm^3^) following RYGB using Data Analysis Facilitation Suite (DAFS), an automated computed tomography (CT) analysis software.

**Methods:**

In this prospective pilot study, nine female patients underwent low-dose abdominal and pelvic CT imaging at baseline, 3-, and 6-months post-RYGB. Volumetric analysis from the ninth thoracic veterbra (T9) to the sacrum was performed using DAFS. Changes in SM, VAT, and SAT were assessed using paired t-tests.

**Results:**

Participants (mean ± SD; age 35 ± 9 years, BMI 48 ± 10 kg/m²) experienced substantial weight loss (14 ± 5% at 3 months, 25 ± 7% at 6 months; *p* < 0.001). SAT and VAT volumes decreased significantly by 21% and 27% at 3 months, and by 31% and 47% at 6 months, respectively (all *p* < 0.001). In contrast, SM volume showed a significant decline of 14% at month 3 (*p* < 0.001) and then plateaued thereafter.

**Conclusion:**

The changes over time differ substantially among SM, VAT and SAT after Roux-en-Y gastric bypass, reflecting the distinct physiological responses and metabolic improvement of different tissue types. Larger and longer clinical studies are needed to validate these findings.

## Key points

• RYGB leads to large relative reductions in visceral and subcutaneous adipose tissue (VAT and SAT) volumes at 6 months while skeletal muscle (SM) volume shows a more modest relative decline at 3 months and then stabilizes at 6 months.

• There are meaningful correlations between SM and SAT, whereas the correlations between VAT and SAT, and between SM and VAT, appear weak or negligible.

• Findings suggest RYGB is associated with skeletal muscle preservation relative to fat loss, but larger, longer-term studies are needed to confirm these results and their clinical implications.

## Introduction

Metabolic and bariatric surgery (MBS) is the most effective treatment for obesity and its related comorbidities [1]. Patients treated with MBS typically experience marked weight loss within the first 3–6 months, reaching a nadir between 12 and 24 months, with long-term weight loss averaging 25–35% of initial body weight depending on the operative procedure. Notably, > 80% of this loss in mass is attributed to fat, underscoring the importance of understanding body composition changes post-MBS [2–5].

Several clinical trials have explored body composition changes following various MBS operations, linking these shifts particularly in adipose tissue depots to the postoperative metabolic improvements [3–5]. Given the putative physiologic roles of visceral and subcutaneous adipose tissues (VAT and SAT) in these outcomes, precise pre- and post-operative body composition assessment critical [4, 5]. Numerous studies have explored how different MBS procedures affect body composition and metabolic outcomes; however, common and convenient methods (e.g. bioelectrical impedance analysis (BIA), dual-energy X-ray absorptiometry (DXA) have their limitations. BIA underestimates VAT in individuals with high VAT [6], and DXA may overestimate lean soft tissue changes by over 10% compared to CT following MBS [7]. Although CT imaging offers superior accuracy, its use in MBS studies has been limited. Most CT-based studies have focused on VAT reduction in relation to weight loss and metabolic gains [8–10], and often rely on single-slice or short-sequence protocols that may miss broader changes in body composition [6, 7]. Additionally, there is a lack of special consideration for quantitative and qualitative muscle mass analysis using gold standards for body composition assessment. This is particularly important considering the role of skeletal muscle (SM) tissue in lifespan functionality and health [11].

Accurate tracking of serial body composition changes after MBS is valuable and can help clinicians identify causes of suboptimal outcomes to ultimately improve patient care. In this study, we aimed to evaluate short-term changes in body composition following Roux-en-Y gastric bypass (RYGB) using the Data Analysis Facilitation Suite (DAFS). DAFS is an on-site automated medical imaging analytics platform for CT, MRI, and PET imaging that performs precise anatomical segmentation from single slices to full 3D whole-body scans without external data transfer. It quantifies all major fat depots, skeletal muscle groups, and key organs to generate standardized, reproducible body-composition biomarkers. DAFS also integrates PET tracer-uptake analysis within anatomically defined regions, providing detailed metabolic and oncologic assessment. With high-quality 3D visualization and a simple keyword-based interface for extracting metrics from single images or large datasets, DAFS enables clinical researchers to easily transform routine imaging into actionable insights for metabolic risk stratification, cancer evaluation, longitudinal monitoring across disease, aging, and interventions [12–17].

DAFS supports multiple clinical applications by (1) enabling metabolic disease assessment through quantification of visceral, subcutaneous, and ectopic fat for risk stratification in obesity, diabetes, and metabolic dysfunction associated fatty liver disease; (2) augmenting oncology workflows by integrating PET tracer-uptake within anatomically segmented regions to quantify metabolic tumor burden and by profiling sarcopenia and adiposity; (3) supporting liver and abdominal disease evaluation with organ volumetry and fat quantification to assess steatosis and hepatomegaly; and (4) enabling aging and frailty assessment through automated skeletal-muscle quantification for sarcopenia and longitudinal tracking of body-composition changes [12–17].

## Methods

### Patient selection

This prospective pilot study was approved by the Pennington Biomedical Research Center Institutional Review Board. Adults (BMI ≥ 30 kg/m²) evaluated for laparoscopic RYGB were eligible. Only those deemed suitable surgical candidates were invited to participate. Participants were excluded for pregnancy, inflammatory bowel disease, chronic kidney disease, significant liver disease, uncontrolled thyroid disorders, prior bowel resection, excessive radiation exposure, or any condition impairing PET/CT scan tolerability.

### Study design

The main study’s primary endpoint was measurement of intestinal glucose uptake using PET/CT imaging, which is reported elsewhere [18]. Of the nine enrolled participants, one relocated > 200 miles before the 6-month scan. The remaining eight completed all imaging at baseline, and at 3 and 6 months postoperatively (± 10 days). Preoperative CT imaging was performed one month before surgery, prior to initiating a short-term low-calorie liquid diet, which is part of routine preoperative management.

### Imaging acquisition and analysis

Low-dose CT imaging of the abdomen and pelvis was performed as part of an ^18^F-FDG PET/CT scan using the Discovery IQ system (GE Healthcare) at Mary Bird Perkins Cancer Center, Baton Rouge, LA. CT images were analyzed using the Data Analysis Facilitation Suite (DAFS v3) Voronoi Health Analytics (https://www.voronoihealthanalytics.com). Regions of interest (ROIs) were delineated on baseline and postoperative scans using DAFS software. A nonlinear algorithm segmented multi-slice, multi-tissue structures and labeled axial slices by vertebral level. The abdominal adipose tissues and skeletal muscle volumes (cm³) were quantified from the ninth thoracic veterbra (T9) to the sacrum. All automated segmentations and vertebral-level labels were manually reviewed and corrected by ZNH to ensure anatomical accuracy. This included verifying the correct identification of T9 and the sacrum, correcting any mis-segmented adipose or muscle boundaries, and standardizing across baseline and postoperative scans.

### Operative technique and patient preparation

All participants underwent a standard proximal RYGB using a standard approach. The jejunum was divided ~ 100 cm distal to the ligament of Treitz, and a 125 cm Roux limb was anastomosed to the biliopancreatic limb via stapled jejunojejunostomy. The Roux limb was advanced antecolic to the stomach, where a 30 ml gastric pouch was created and connected via a hand-sewn, end-to-end gastrojejunostomy and the mesenteric defects were routinely closed.

Standard preoperative preparation includes comprehensive support, such as educational materials on diet, exercise, and behavioral strategies delivered during consultations and reinforced throughout clinic visits. Patients receive dietary counseling from dietitians or obesity medicine specialists, individualized exercise plans with measurable goals, and behavioral support through lifestyle change counseling and adherence strategies during the postoperative follow up visits. In the postoperative period, patients are advised to follow a low-calorie, high-protein diet along with bariatric multivitamin supplementation. Activity restrictions are generally limited to avoiding heavy lifting for the first month, after which gradual progression to regular physical activity and strengthening exercises is encouraged.

### Statistical analysis

Percentage total weight loss (%TWL) was calculated as [(operative weight – follow-up weight)/operative weight)] × 100. Remission of diabetes was defined as follows: HbA1c level < 6.5% or FBG < 7.0 mmol/L (126 mg/dL) without the use of an oral hypoglycemic agent or insulin therapy [19].

Baseline demographics, anthropomorphic, comorbidity measurements, body mass index (BMI), and weight loss were examined over time. Continuous variables were summarized as mean ± standard deviation (SD), while categorical variables were presented as counts and frequencies. The p-value reflects the change from baseline to 3 months and from baseline to 6 months. A linear mixed-effects model was used to estimate changes at 3 and 6 months relative to baseline, accounting for the correlation among repeated measurements within subjects. To assess whether the change followed a linear pattern, a quadratic term was added to the model; a significant quadratic term indicated deviation from linearity. To compare change patterns among different body-composition components, a mixed model was used to account for correlations among repeated measurements within subjects and across muscle and fat compartments. All analyses were performed using R statistical software (Version 4.5.0).

## Results

### Patient characteristics

Of the nine female participants (mean age 35 ± 9 years), seven underwent primary RYGB for severe obesity, and two underwent conversion RYGB from a prior sleeve gastrectomy due to weight recurrence. Five participants were White/Caucasian, and four were Black/African American. Mean preoperative weight was 121 ± 29 kg and BMI 48 ± 10 kg/m². Comorbidities included type 2 diabetes (T2D, *n* = 4), hypertension (*n* = 4), biopsy-confirmed MASH (*n* = 3), and dyslipidemia (*n* = 1). No 30-day complications, readmissions, reinterventions, or reoperations occurred. At 6 months after RYGB, all patients with T2D had 100% diabetes resolution (Table 1).

**Table 1:** Patient characteristics

Patient #					Preoperative					6 months Postoperative				
	Age, years	Preop HT	Preop HL	Preop T2D	A1C, (%)	T Chol, (mg/dL)	TG, (mg/dL)	HDL, (mg/dL)	LDL, (mg/dL)	A1C, (%)	T Chol, (mg/dL)	TG, (mg/dL)	HDL, (mg/dL)	LDL, (mg/dL)
1	30	Yes	No	No	5.3	173	134	49	97	na	na	na	na	na
2	38	No	No	No	5.6	184	64	51	121	4.8	na	na	na	na
3	34	Yes	No	Yes, non-insulin	5.6	227	146	47	154	5.4	174	124	45	107
4	55	Yes	Yes	Yes, insulin	7.3	110	90	50	43	5.9	na	na	na	na
5	31	No	No	No	5.6	150	69	49	87	5.3	127	37	51	66
6	33	No	No	No	5.2	264	129	47	194	na	192	91	46	129
7	31	No	No	No						4.9	140	67	48	78
8	25	No	No	Yes, non-insulin	5.5	137	61	34	90	na	na	na	na	na
9	40	Yes	No	Yes, non-insulin	6.7	164	109	42	102	4.8	na	na	na	na

*HT*-hypertension, *HL*- hyperlipidemia, *T2D*- type 2 diabetes, Preop-preoperative, *A1C*-Glycated hemoglobin, *T Chol*- total cholesterol, *TG*- triglyceride, *HDL*-high-density lipoprotein, *LDL*- low-density lipoprotein

### Weight loss and body composition outcomes

**Fig. 1:**
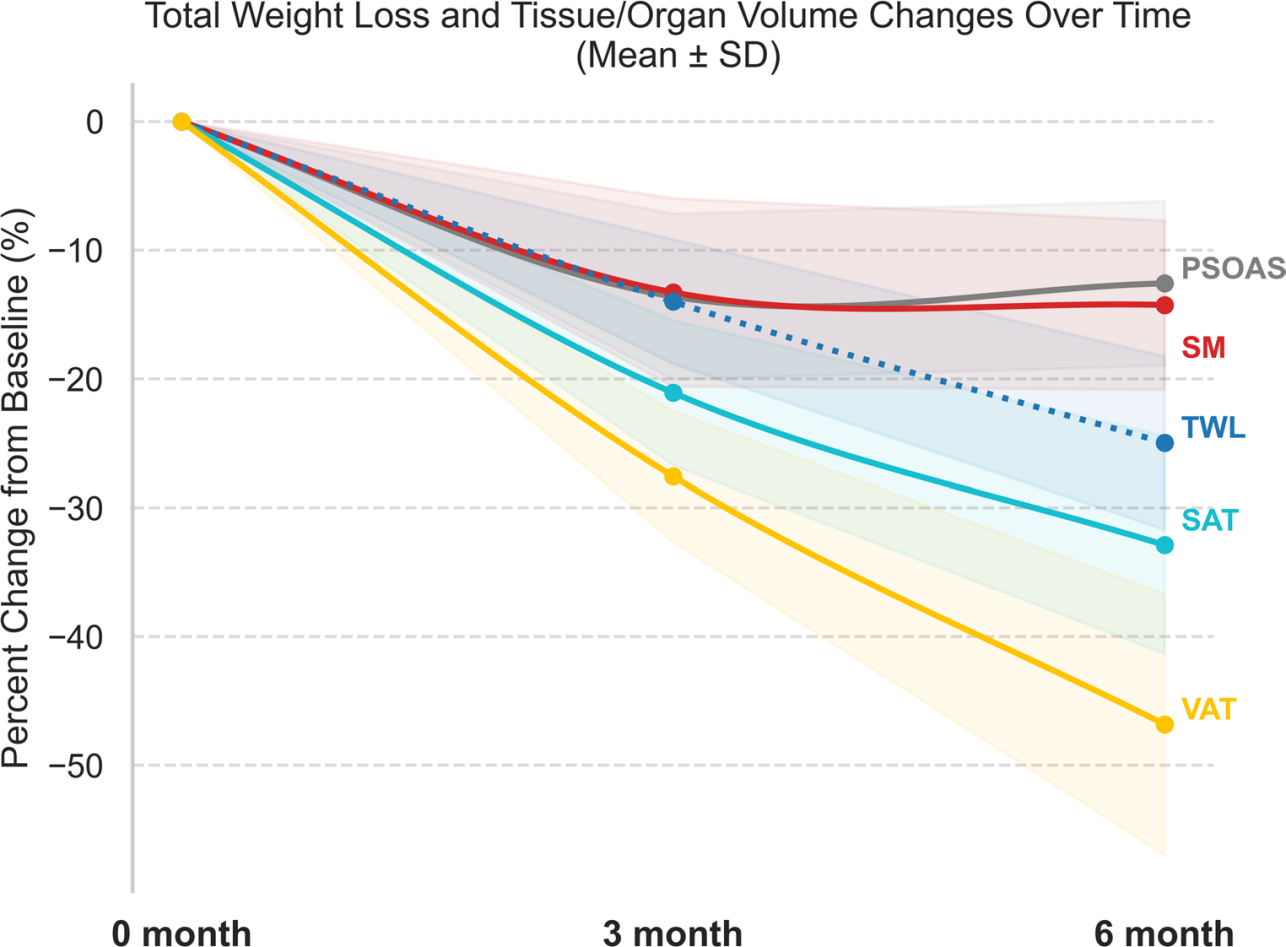
Mean percentage changes of total weight loss and tissue volumes at 3- and 6-months after Roux-en-Y gastric bypass

All patients demonstrated substantial weight loss, averaging 14 ± 5% at 3 months and 25 ± 7% at 6 months (*p* < 0.001). Figure 1A illustrates the trajectory of TWL% across the 9 patients. Table 2 provides a detailed summary of each patient’s weight, SAT, VAT, SM volumes at each time point.

**Table 2:** Serial anthropometric and tissue volume changes over time

#Patient	Body Weight (kg)			BMI (kg/m^2^)			Total Wt Loss %		SM Volume (cm^3^)			Psoas Volume (cm^3^)			VAT Volume (cm^3^)			SAT Volume (cm^3^)		
	Pre op	3 M	6 M	Pre op	3 M	6 M	3 M	6 M	Pre op	3 M	6 M	Pre op	3 M	6 M	Pre op	3 M	6 M	Pre op	3 M	6 M
**1**	90	72	62	46	37	32	21	31	4287	3446	3496	486	389	405	2864	1833	1150	17,637	12,653	10,766
**2**	99	91	82	34	31	28	8	18	6816	6245	6096	744	683	681	2318	1673	946	17,295	14,198	11,774
**3**	138	116	107	48	40	37	16	23	6470	5796	5943	672	602	620	5109	4023	3379	23,522	21,032	19,926
**4**	88	81	75	33	31	28	8	15	4865	4468	4248	481	452	434	4478	3312	2510	12,261	9720	8963
**5**	136	111	97	55	45	39	18	29	6271	4700	4732	763	564	579	2768	1964	1577	28,867	22,676	18,573
**6**	108	99	86	44	40	35	9	21	5229	4920	4844	611	559	555	2884	2122	1777	21,955	15,805	14,177
**7**	160	139	112	62	54	44	13	30	7248	5647	5646	855	666	679	3728	2939	2233	26,085	19,887	16,541
**8**	165	143	na	57	49		13	na	7264	6322	na	874	700	na	2411	1785	na	34,132	28,587	na
**9**	108	87	72	51	42	34	19	33	4413	4162	3963	464	430	433	5304	3484	2323	18,224	14,087	10,517

*Pre-op*- preoperative, *M*- month, *BMI*-body mass index, *SM*-skeletal muscle, *SAT*-subcutaneous adipose tissue, *VAT*-visceral adipose tissue, *Wt*-weight

**Fig. 2:**
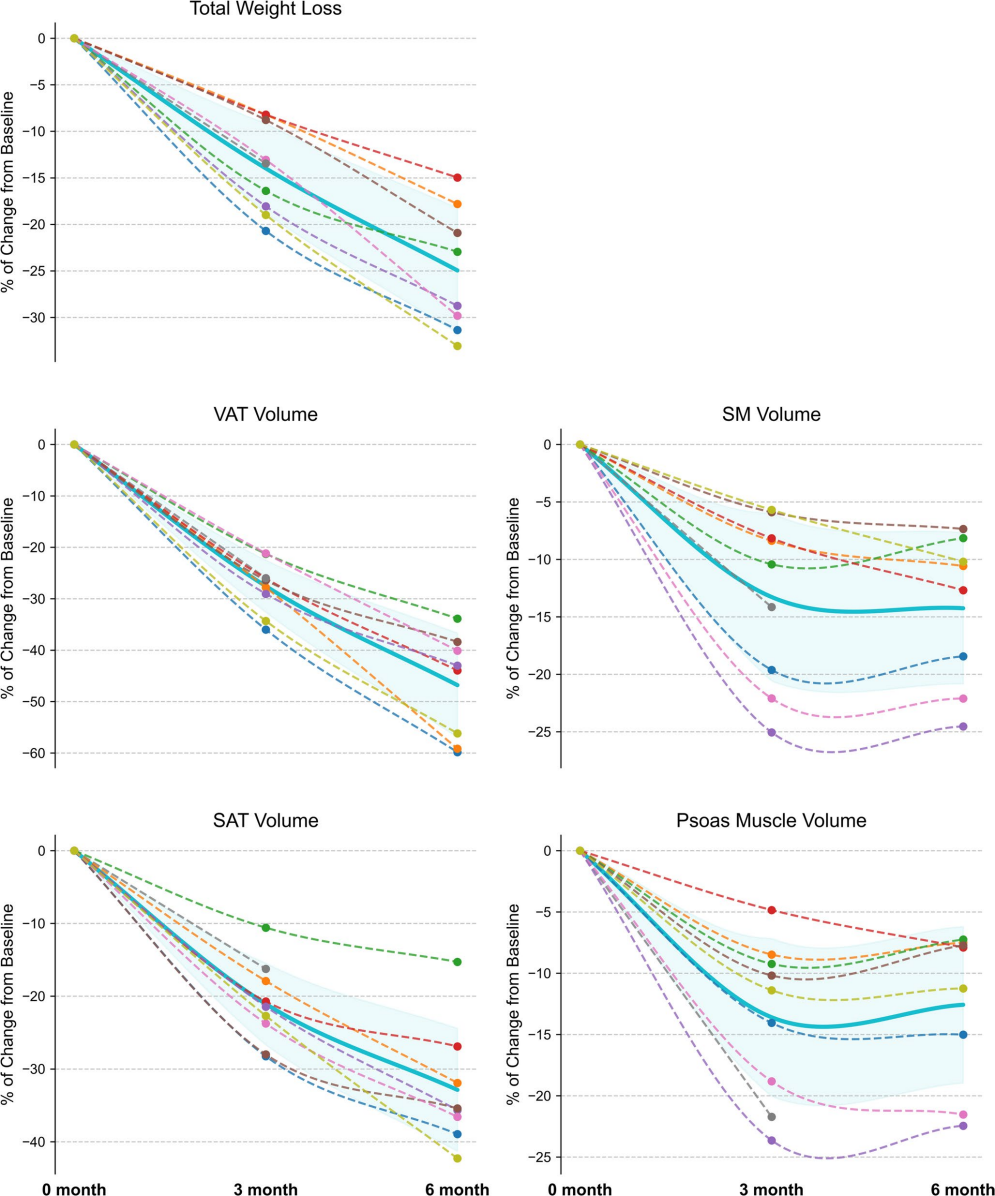
Longitudinal changes in total weight loss and body composition following Roux-en-Y gastric bypass Percentage changes over time are shown for: (**A**) total weight loss, (**B**) visceral adipose tissue volume, (**C**) subcutaneous adipose tissue volume, (**D**) skeletal muscle volume, and (**E**) psoas muscle volume. Dotted lines: Individual patient trajectories (each color represents individual patient). Solid line with shaded area: Mean trajectory with SD

The percentage changes in mean TWL and tissue volumes are shown in Fig. 2, highlighting that the weight loss following RYGB was primarily driven by reductions in SAT and VAT. There was a significant reduction of both SAT and VAT after RYGB. SAT volume decreased by 21 ± 6% at 3 months and 31 ± 8% at 6 months (*p* < 0.001) and VAT volume decreased by 27 ± 5% at 3 months and 47 ± 10% at 6 months (*p* < 0.001). Figures 1B-E illustrate the individual trajectories of SAT, VAT, SM and psoas muscle volume changes over time after RYGB.

For SAT specifically, the decrease showed a trend toward deviation from a linear pattern (*p* = 0.053), with a rapid decline before month 3 followed by a substantially slower decline afterward. In contrast, reductions in BW and VAT did not deviate from a linear pattern. Meanwhile, the change in SM deviated significantly from a linear trend (*p* = 0.007): it showed a significant 14% decline at month 3 and then plateaued thereafter (Table 3).

**Table 3:** Longitudinal change of body composition components

Measure	Month	Estimate	*P* value (overall change)	*P* value (linear change)
**BW**	3	−0.124	< 0.001	0.396
	6	−0.248		
**SM**	3	−0.135^#^	< 0.001	0.007
	6	−0.144^#^		
**VAT**	3	−0.237	< 0.001	0.292
	6	−0.473		
**SAT**	3	−0.157	< 0.001	0.053
	6	−0.315		

*BW* = Body Weight, *SM* = Skeletal Muscle, *VAT* = Visceral Adipose Tissue, *SAT* = Subcutaneous Adipose Tissue. ^#^ A quadratic term was included

**Fig. 3:**
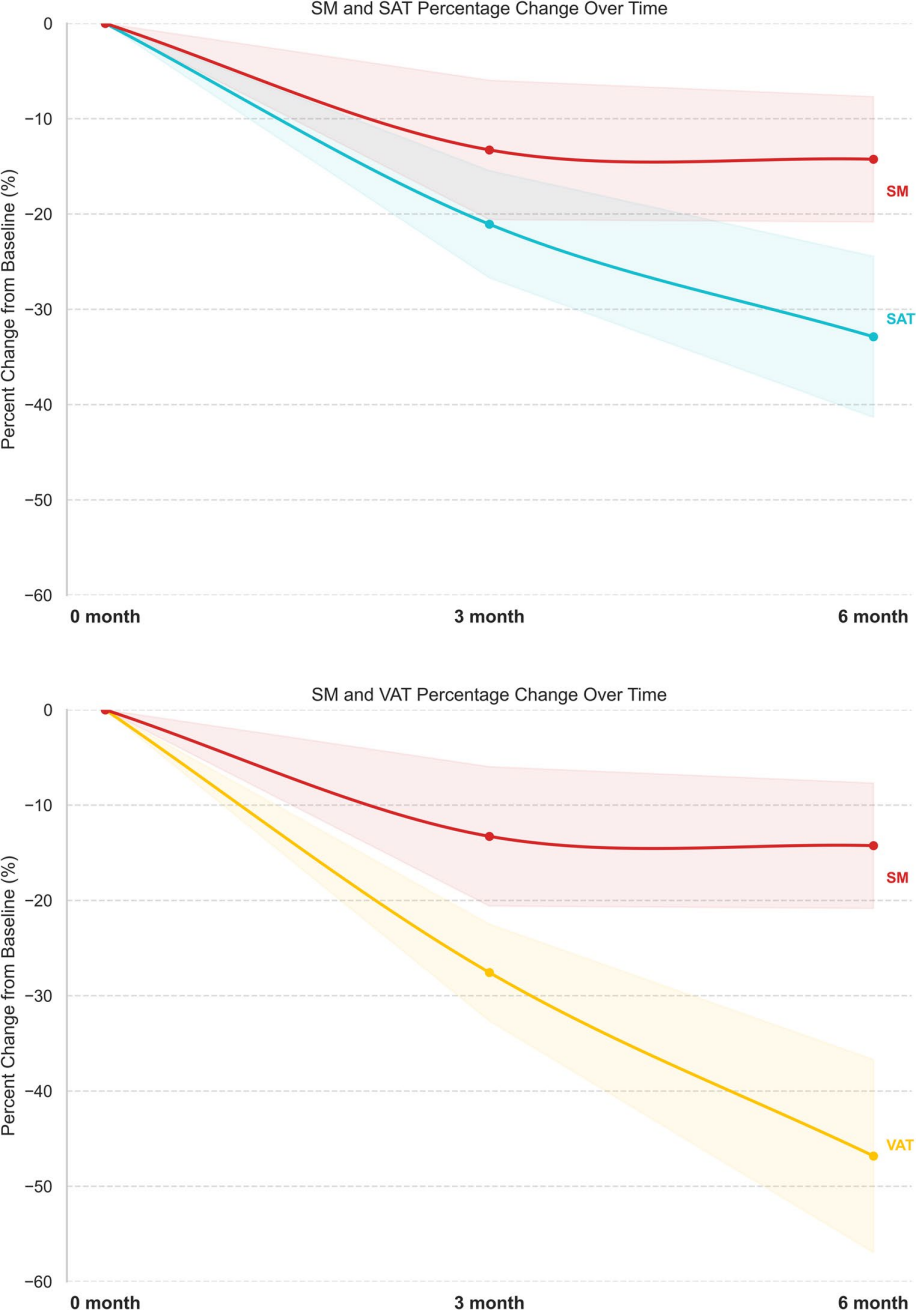
Comparison of percentage changes following Roux-en-Y gastric bypass; Skeletal muscle (SM) vs. Subcutaneous adipose (SAT) and Visceral adipose tissue (VAT)

Both SAT and VAT showed faster reduction compared to SM. At month 6, decreases in SAT and VAT were significantly greater than those in SM. At month 3, the difference in reduction between SM and SAT was not statistically significant, likely due to small sample size (Table 4; Fig. 3).

**Table 4:** The differential % change rate between skeletal muscle and fat tissues (SAT and VAT)

Month	Δ Change rate (%) SAT vs. SM	Δ Change rate (%) VAT vs. SM
**3**	−7.10	−13.9*
**6**	−16.8*	−32.7*

*SM* Skeletal Muscle, *VAT* Visceral Adipose Tissue, *SAT* Subcutaneous Adipose Tissue

**Fig. 4:**
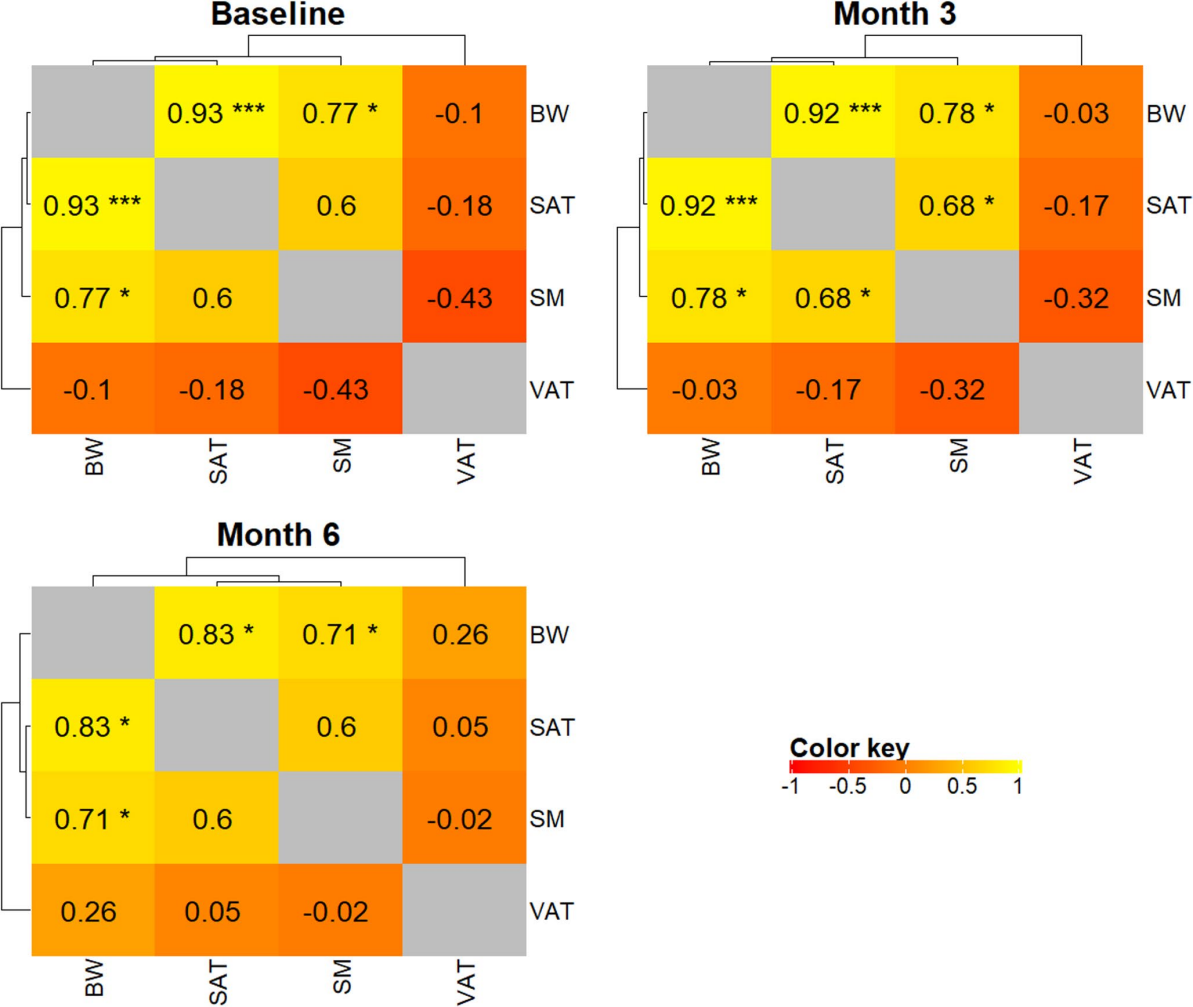
Correlation heatmaps at different time points (baseline, 3- and 6-months after surgery) to examine the relationships among changes in muscle and fat compartments from a complementary perspective

Due to the pronounced effects of RYGB on body composition, we generated correlation heatmaps at different time points to examine the relationships among changes in muscle and fat compartments from a complementary perspective (Fig. 4). The correlations between BW and SM were around 0.78 at baseline and month 3, while the correlations between BW and SAT were around 0.92 at these time points. By month 6, these correlations decreased to 0.71 (BW vs. SM) and 0.83 (BW vs. SAT). These results indicate that BW and SM/SAT were tightly linked before and early after surgery; however, by month 6, these relationships weakened, reflecting potentially increased inter-individual heterogeneity and differential change patterns among muscle and fat compartments, thereby reducing the linear association between BW and specific tissues – BW is a weaker predictor of fat tissues.

### Serial CT volumetrics

Changes in SAT and VAT were captured using 3D CT imaging, automated through the DAFS software. A sample of serial CT scans from patient 1 illustrates the progression of.

**Fig. 5:**
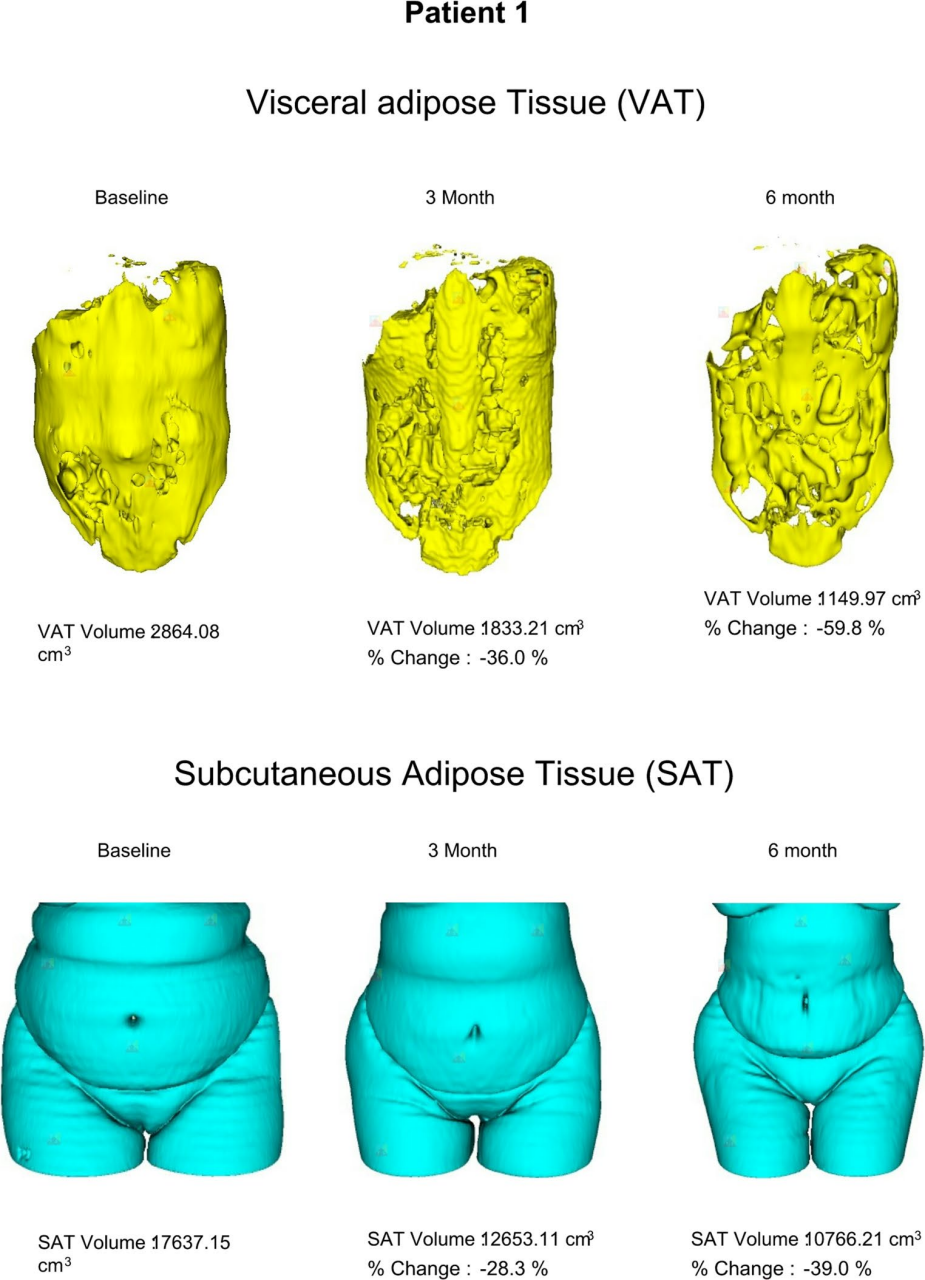
Serial CT images illustrating longitudinal changes in adipose tissue volumes for Patient 1: (**A**) Visceral adipose tissue volume and (**B**) Subcutaneous adipose tissue

SAT (Fig. 5A**)** and VAT (Fig. 5B) change at various time points following RYGB. Patient 1 demonstrated the greatest reduction in VAT volume with TWL of 31% at 6 months after RYGB, as shown in Table 2.

## Discussion

This prospective pilot study demonstrated feasibility of the DAFS automated CT segmentation to monitor body tissue volumes over time in patients undergoing MBS. The weight loss secondary to surgical obesity treatment was associated with large relative reductions in SAT (33%) and VAT (47%) volumes within six months postoperatively. In addition to adiposity changes, there was a relatively small, non-significant trend (13–14%) in SM volume loss within the psoas muscle despite the TWL of 25% at 6 months post RYGB. These findings align with prior literature indicating that fat mass loss accounts for the majority of early postoperative weight reduction following MBS [1–3].

In an era of highly effective obesity treatments, understanding body composition changes during rapid weight loss is becoming increasingly important. A meta-analysis [20] evaluated time-dependent changes in lean body mass (LBM, *n* = 37), fat-free mass (FFM, *n* = 20), and skeletal muscle mass (SMM, *n* = 3) after MBS. The majority of these longitudinal assessments were carried out using DXA (*n* = 56) and MRI (*n* = 3). At 12 months post-surgery, average losses were 3 kg of SMM, 8 kg of FFM and LBM, which reflects 21% and 22% of TWL. About 55% of the LBM loss occurred within the first 3 months, with similar trends for FFM and SMM, suggesting that a large proportion of FFM, LBM and SMM loss occurs 3 months after surgery [20, 21].

Body fat distribution plays a significant role in the cardiometabolic consequences of obesity. Prior studies have demonstrated associations between visceral fat and cardiometabolic outcomes [7–9, 22]. Galanakis et al. [8] used abdominal CT imaging to evaluate changes in SAT and VAT and their metabolic impact following MBS in 38 patients (18 sleeve gastrectomy, 20 gastric banding). Significant reductions in both SAT and VAT were observed at 6 and 12 months, with VAT reduction being significantly greater at 12 months (*P* < 0.01). The SAT/VAT ratio increased from 4.1 ± 1.7 preoperatively to 6.2 ± 3.1 postoperatively (*P* < 0.001) [8]. Additionally, high-sensitivity C-reactive protein levels declined in association with total abdominal fat loss [8]. Similar patterns were observed in the current cohort, with a 47% reduction in VAT and a 31% reduction in SAT at six months post-RYGB. Notably, all patients achieved complete remission of type 2 diabetes. These findings of the lower SAT: VAT ratio indicate a disproportionately higher visceral fat burden and thus an elevated metabolic risk. This further supports the association between visceral fat reduction, enhanced insulin sensitivity, and improved cardiometabolic outcomes, even within a small sample size. In our cohort, SAT and VAT volumes decreased substantially by 21% and 27% at 3 months, and by 31% and 47% at 6 months, respectively (all *p* < 0.001). The SM volume showed a modest but non-significant decline, 13% at 3 months and 14% at 6 months post-RYGB. Notably, four patients (44%) demonstrated recovery in SM volume following the initial decrease at 3 months, as illustrated in Fig. 5. This is likely due to muscle adaptation, and the measurements were taken from truncal muscles because the CT scan was performed on the abdominal region.

Muscle mass reduction is now understood to be a significant component of weight loss achieved with GLP-1 therapies [23], and this loss may contribute to the elevated risk of fractures after MBS by impairing muscular strength and postural stability. This highlights the need to monitor muscle loss, understand its mechanisms, and explore potential preventive interventions. The mechanisms underlying the SM volume decline following MBS are multifactorial. Primarily, the reduction in dietary protein due to decreased caloric intake (500–800 kcal/day) that can occur postoperatively may make it challenging to meet recommended protein intake (e.g. 60 g/day or 1.1 g/kg ideal body weight) [21, 24–26]. Inadequate protein intake leads to negative nitrogen balance and muscle breakdown to meet metabolic demands [27]. Second, limited physical activity, especially restriction of resistance training for up to six weeks post-surgery to prevent wound complications, likely further contributes to SM loss [25]. Third, marked postoperative weight loss is associated with decreased skeletal loading. While chronic loading secondary to excess body weight led to increased bone density and SM hypertrophy, weight loss is associated with physiologic decreases in SM and bone mass. This long-term SM loss could result in reduction in muscle strength, which may contribute to increased risk of frailty, functional disability, and mortality. Therefore, emphasizing the importance of early perioperative strategies to preserve muscle mass after MBS is essential [20, 25]. Additionally, the results also indicate that with targeted nutritional intervention and structured physical activity, preservation of muscle during early postoperative phase is achievable. Further longer-term studies are needed to evaluate the risk of sarcopenia following substantial weight loss.

Monitoring body composition changes, particularly in muscle and fat tissues after MBS remains challenging. Sylivris et al. [4] reported mean fat mass reductions range from 21 to 27 kg, while lean mass changes vary widely, from 4% to 17% at one-year post-RYGB. This variability is likely due to differences in measurement techniques, as commonly used methods like BIA and DXA have limitations in accuracy, especially in patients with higher BMI. DXA, while widely used, may overestimate lean mass changes compared to CT, and BIA tends to underestimate visceral fat in individuals with high adiposity.

To date, no randomized trials have specifically evaluated lean and fat mass changes in MBS patients using CT imaging. Existing CT-based studies are largely observational and tend to focus on VAT reduction or metabolic improvements, often relying on single-slice or limited-sequence protocols [4, 20]. Our study partially addresses this gap by employing longitudinal, multi-slice CT analysis with automated segmentation via the DAFS to assess volumetric changes in skeletal muscle and adipose tissue. The use of DAFS-enabled CT segmentation allowed for precise tracking of body composition changes from the selected vertebrae, T9 to the sacrum. CT imaging offers superior accuracy in evaluating regional abdominal tissues, particularly in individuals with higher BMI, compared to common and traditional methods such as BIA and DXA. These conventional techniques are limited by weight sensitivity and reduced precision in quantifying visceral fat and lean mass [4–6]. Given that abdominal CT is one of the most performed imaging modalities in hospital settings, particularly when patients with obesity present with abdominal symptoms, this may represent an opportunity to repurpose existing clinical scans to assess body composition in individuals with obesity. Our study demonstrates the feasibility of using longitudinal, multi-slice CT imaging with automated DAFS segmentation to quantify changes in skeletal muscle and adipose tissue volumes, contributing to a more precise understanding of postoperative body composition changes in MBS patients.

Despite the small sample size and short follow-up, our findings contribute to the growing evidence emphasizing the importance of body composition measurement and monitoring in obesity and MBS patients [28]. The absence of 30-day complications and the consistent imaging protocol strengthen the internal validity of our specific analysis on body compositions after MBS. However, the study has notable limitations, including the lack of postoperative metabolic data for all participants and the exclusion of male patients, which may affect generalizability. As this was a secondary analysis of CT imaging originally conducted for a gut metabolism study, blood samples and other measurements (i.e. grip strength) were not included in the scope of the original project, limiting our ability to correlate imaging findings with biochemical or functional markers Nevertheless, while the sample is small, the statistical findings provide directional insight and may help guide future study design and power calculations.

## Conclusion

Serial CT imaging with automated DAFS segmentation is a feasible approach to track changes in body composition after MBS, demonstrating consistent reductions in VAT and SAT and modest but partially recoverable muscle loss. Although limited by small sample size and preliminary data, these findings offer useful directional insight and support the value of CT-based analysis in postoperative monitoring in MBS patients. Larger, longer-term studies are needed to clarify trajectories of lean and fat mass and to guide strategies for muscle preservation after MBS.

## Data Availability

No datasets were generated or analysed during the current study.
